# 5-[(*E*)-(2-Hy­droxy­benzyl­idene)amino]-1*H*-1,3-benzimidazole-2(3*H*)-thione

**DOI:** 10.1107/S160053681005172X

**Published:** 2010-12-18

**Authors:** Zishan Tabassum, Othman Sulaiman, Mohd. Afzal, Madhukar Hemamalini, Hoong-Kun Fun

**Affiliations:** a School of Industrial Technology, Universiti Sains Malaysia, 11800 USM, Penang, Malaysia; b Department of Chemistry, Aligarh Muslim University, Aligarh U. P. 202 002, India; c X-ray Crystallography Unit, School of Physics, Universiti Sains Malaysia, 11800 USM, Penang, Malaysia

## Abstract

There are two mol­ecules in the asymmetric unit of the title compound, C_14_H_11_N_3_OS. In each, the benzimidazole ring system is essentially planar, with maximum deviations of 0.010 (2) and 0.006 (2) Å, and makes dihedral angles of 8.70 (9) and 13.75 (8)°, respectively, with the hy­droxy-substituted benzene rings. Each mol­ecule adopts an *E* configuration about the central C=N double bond. In the crystal, the two independent mol­ecules are connected *via* inter­molecular N—H⋯S hydrogen bonds, forming dimers. Furthermore, the dimers are connected by N—H⋯O hydrogen bonds into mol­ecular ribbons along the *c* axis. There is an intra­molecular O—H⋯N hydrogen bond in each mol­ecule, which generates an *S*(6) ring motif.

## Related literature

For applications of benzimidazole compounds, see: Grassmann *et al.* (2002 ▶); White *et al.* (2004 ▶); Demirayak *et al.* (2002 ▶). For hydrogen-bond motifs, see: Bernstein *et al.* (1995 ▶).
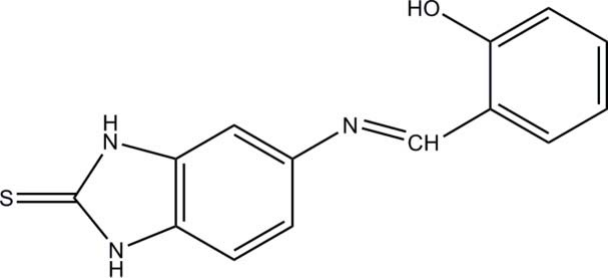


## Experimental

### 

#### Crystal data

C_14_H_11_N_3_OS
*M* *_r_* = 269.32
Monoclinic, 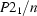

*a* = 8.2680 (2) Å
*b* = 28.1043 (6) Å
*c* = 10.5047 (2) Å
β = 92.234 (1)°
*V* = 2439.08 (9) Å^3^
*Z* = 8
Mo *K*α radiation
μ = 0.26 mm^−1^
*T* = 296 K
0.44 × 0.28 × 0.05 mm

#### Data collection

Bruker SMART APEXII CCD area-detector diffractometer
Absorption correction: multi-scan (*SADABS*; Bruker, 2009 ▶) *T*_min_ = 0.894, *T*_max_ = 0.986
27759 measured reflections
7101 independent reflections
4852 reflections with *I* > 2σ(*I*)
*R*_int_ = 0.064

#### Refinement

*R*[*F* ^2^ > 2σ(*F* ^2^)] = 0.063
*wR*(*F* ^2^) = 0.140
*S* = 1.06
7101 reflections
367 parameters
H atoms treated by a mixture of independent and constrained refinement
Δρ_max_ = 0.53 e Å^−3^
Δρ_min_ = −0.37 e Å^−3^

### 

Data collection: *APEX2* (Bruker, 2009 ▶); cell refinement: *SAINT* (Bruker, 2009 ▶); data reduction: *SAINT*; program(s) used to solve structure: *SHELXTL* (Sheldrick, 2008 ▶); program(s) used to refine structure: *SHELXTL*; molecular graphics: *SHELXTL*; software used to prepare material for publication: *SHELXTL* and *PLATON* (Spek, 2009 ▶).

## Supplementary Material

Crystal structure: contains datablocks global, I. DOI: 10.1107/S160053681005172X/rz2535sup1.cif

Structure factors: contains datablocks I. DOI: 10.1107/S160053681005172X/rz2535Isup2.hkl

Additional supplementary materials:  crystallographic information; 3D view; checkCIF report

## Figures and Tables

**Table 1:** Hydrogen-bond geometry (Å, °)

*D*—H⋯*A*	*D*—H	H⋯*A*	*D*⋯*A*	*D*—H⋯*A*
N1*A*—H1*NA*⋯S1*B*^i^	0.86 (3)	2.61 (3)	3.4714 (19)	173 (3)
N2*A*—H2*NA*⋯O1*B*^ii^	0.86 (3)	1.99 (3)	2.781 (2)	153 (3)
N1*B*—H1*NB*⋯O1*A*^iii^	0.87 (2)	2.16 (2)	2.936 (2)	149 (2)
N2*B*—H2*NB*⋯S1*A*^iv^	0.84 (3)	2.45 (3)	3.2547 (19)	163 (2)
O1*B*—H1*OB*⋯N3*B*	0.99 (4)	1.64 (3)	2.552 (2)	152 (3)
O1*A*—H1*OA*⋯N3*A*	0.99 (4)	1.72 (4)	2.600 (3)	147 (3)

Symmetry codes: (i) 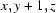; (ii) 
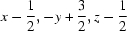
; (iii) 
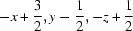
; (iv) 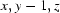.

**Fig. 1.**
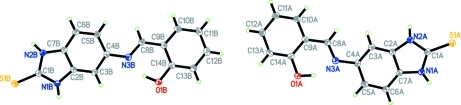
The asymmetric unit of the title compound, showing 50% probability displacement ellipsoids and the atom-numbering scheme.

**Fig. 2.**
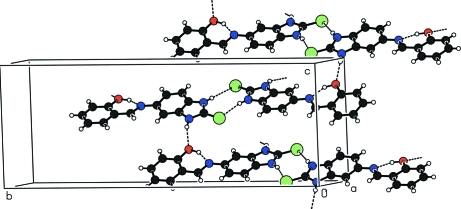
A molecular ribbon generated by N—H···O hydrogen bonds.

## supplementary crystallographic information

### Comment

Benzimidazole and its derivatives are very important for the development of molecules of pharmaceutical and biological interest.  Schiff bases play an important role in bioorganic chemistry as they exhibit remarkable antihistamine (Grassmann *et al.*, 2002),  antitumour (White *et al.*, 2004) and potential anticancer activities (Demirayak *et al.*, 2002).  In view of their importance in the field of drug discovery, the crystal structure determination of the title compound was carried out and the results are presented here.

The asymmetric unit of title compound which contains two molecules [A & B] is shown in Fig. 1. All geometrical parameters are within normal ranges. Each molecule adopts an *E* configuration about the central C═N double bond.  For both molecules, the benzimidazole ring systems (N1A–N2A/C1A–C7A)/(N1B–N2B/C1B–C7B) are essentially planar with a maximum deviation of 0.010 (2) and 0.006 (2) Å, respectively, for atom C7A and C3B. The dihedral angles between the benzimidazole ring system ((N1A–N2A/C1A–C7A)/(N1B–N2B/C1B–C7B) with the hydroxy substituted benzene ring (C9A–C14A)/(C9B–C14B) are 8.70 (9)° and 13.75 (8)° respectively.

In the crystal structure (Fig. 2), the two independent molecules are connected *via* intermolecular N—H···S hydrogen bonds to form dimers (Table 1).  Furthermore, the dimers are connected *via* N—H···O hydrogen bonds to form molecular ribbons along the *c*-axis. There is an intramolecular N—H···O hydrogen bond which generates an *S*(6) (Bernstein *et al.*, 1995) ring motif in each molecule.

### Experimental

The title compound was synthesized by adding salicyaldehyde (0.122 g, 1 mmol; Alfa Aesar) to a stirred methanolic solution (20 ml) of 5-amino-2-mercaptobenzimidazole (0.165g, 1mmol; Sigma) in a 1:1 molar ratio. The reaction mixture was stirred for half an hour at room temperature. The yellow precipitate which formed was filtered off under vaccum, washed thoroughly with ice-cold methanol and dried in vacuo over anhydrous CaCl_2_ (yield: 73%). Single crystals suitable for X–ray diffraction analysis were obtained from recrystallisation of the Schiff Base in a mixture of DMF:ethanol (95:5 *v*/*v*). M. p, 456–458K. Anal. Calcd. for C_14_H_11_N_3_SO (%) C, 62.44; H, 4.11; N, 15.61, Found: C, 62.48; H, 4.12; N, 15.12. IR (KBr, cm^-1^) 3170 ν (N–H), 3060 ν(O–H), 2364 ν(S–H), 1608 ν(HC═N), 1485 ν(C–N), 747 ν(C–S), UV-vis (ν_max_, nm) in DMF: 320nm; 353nm. ^1^H NMR (400MHz, DMSO-d_6_, δ, ppm): 8.96 (1H, s, HC═N), 13.19 (1H, s, OH), 6.93–6.97 (2H, m, ArH), 7.17–7.40 (4H, m, ArH), 7.62–7.64 (1H, d, ArH) 3.38 (1H, s, SH), 12.62 (z1H, d, NH), ^13^C NMR (100MHz, DMSO–d_6_, δ, ppm): 169.53, 162.53, 160.73, 143.41, 133.60, 133.27, 132.90, 131.97, 119.77, 119.39, 117.36, 116.93, 110.23, 102.07, 79.67, 79.34, 79.01. MS ESI: (m/z) 270.4.

### Refinement

Atoms H1NA, H2NA, H1NB, H2NB, H1OA and H1OB were located from a difference Fourier map and refined freely [N–H = 0.84 (3)–0.87 (3) Å and O–H = 0.99 (4) Å].  The remaining H atoms were positioned  geometrically [C–H = 0.93 Å] and were refined using a riding model, with *U*_iso_(H) = 1.2 *U*_eq_(C).

### Crystal data

**Table:**

C_14_H_11_N_3_OS	*F*(000) = 1120
*M**_r_* = 269.32	*D*_x_ = 1.467 Mg m^−^^3^
Monoclinic, *P*2_1_/*n*	Mo *K*α radiation, λ = 0.71073 Å
Hall symbol: -P 2yn	Cell parameters from 5349 reflections
*a* = 8.2680 (2) Å	θ = 2.4–29.8°
*b* = 28.1043 (6) Å	µ = 0.26 mm^−^^1^
*c* = 10.5047 (2) Å	*T* = 296 K
β = 92.234 (1)°	Plate, yellow
*V* = 2439.08 (9) Å^3^	0.44 × 0.28 × 0.05 mm
*Z* = 8	

### Data collection

**Table:**

Bruker SMART APEXII CCD area-detector diffractometer	7101 independent reflections
Radiation source: fine-focus sealed tube	4852 reflections with *I* > 2σ(*I*)
graphite	*R*_int_ = 0.064
φ and ω scans	θ_max_ = 30.0°, θ_min_ = 2.1°
Absorption correction: multi-scan (*SADABS*; Bruker, 2009)	*h* = −11→11
*T*_min_ = 0.894, *T*_max_ = 0.986	*k* = −32→39
27759 measured reflections	*l* = −14→13

### Refinement

**Table:**

Refinement on *F*^2^	Primary atom site location: structure-invariant direct methods
Least-squares matrix: full	Secondary atom site location: difference Fourier map
*R*[*F*^2^ > 2σ(*F*^2^)] = 0.063	Hydrogen site location: inferred from neighbouring sites
*wR*(*F*^2^) = 0.140	H atoms treated by a mixture of independent and constrained refinement
*S* = 1.06	*w* = 1/[σ^2^(*F*_o_^2^) + (0.0568*P*)^2^ + 0.8644*P*] where *P* = (*F*_o_^2^ + 2*F*_c_^2^)/3
7101 reflections	(Δ/σ)_max_ = 0.003
367 parameters	Δρ_max_ = 0.53 e Å^−^^3^
0 restraints	Δρ_min_ = −0.37 e Å^−^^3^

### Special details

**Table:**

Geometry. All s.u.'s (except the s.u. in the dihedral angle between two l.s. planes) are estimated using the full covariance matrix. The cell s.u.'s are taken into account individually in the estimation of s.u.'s in distances, angles and torsion angles; correlations between s.u.'s in cell parameters are only used when they are defined by crystal symmetry. An approximate (isotropic) treatment of cell s.u.'s is used for estimating s.u.'s involving l.s. planes.
Refinement. Refinement of F^2^ against ALL reflections. The weighted R-factor wR and goodness of fit S are based on F^2^, conventional R-factors R are based on F, with F set to zero for negative F^2^. The threshold expression of F^2^ > 2σ(F^2^) is used only for calculating R-factors(gt) etc. and is not relevant to the choice of reflections for refinement. R-factors based on F^2^ are statistically about twice as large as those based on F, and R- factors based on ALL data will be even larger.

### Fractional atomic coordinates and isotropic or equivalent isotropic displacement parameters (Å^2^)

**Table:**

	*x*	*y*	*z*	*U*_iso_*/*U*_eq_	
S1A	0.21140 (7)	1.12383 (2)	0.02847 (5)	0.02132 (14)	
O1A	0.50964 (18)	0.77807 (6)	0.17652 (15)	0.0221 (4)	
N1A	0.4066 (2)	1.05753 (7)	0.14802 (17)	0.0172 (4)	
N2A	0.2149 (2)	1.02767 (7)	0.02468 (17)	0.0183 (4)	
N3A	0.3807 (2)	0.86052 (7)	0.12424 (16)	0.0176 (4)	
C1A	0.2805 (3)	1.06914 (8)	0.06700 (19)	0.0175 (4)	
C2A	0.2993 (2)	0.98911 (8)	0.07630 (19)	0.0160 (4)	
C3A	0.2787 (3)	0.94091 (8)	0.0614 (2)	0.0184 (5)	
H3AA	0.1966	0.9286	0.0080	0.022*	
C4A	0.3863 (2)	0.91097 (8)	0.12978 (19)	0.0166 (4)	
C5A	0.5118 (2)	0.93050 (8)	0.2077 (2)	0.0189 (5)	
H5AA	0.5835	0.9101	0.2509	0.023*	
C6A	0.5317 (3)	0.97925 (8)	0.2220 (2)	0.0190 (5)	
H6AA	0.6150	0.9918	0.2737	0.023*	
C7A	0.4226 (2)	1.00835 (8)	0.15642 (19)	0.0167 (4)	
C8A	0.2587 (2)	0.83866 (8)	0.07064 (19)	0.0178 (5)	
H8AA	0.1735	0.8563	0.0349	0.021*	
C9A	0.2517 (2)	0.78727 (8)	0.06494 (19)	0.0158 (4)	
C10A	0.1181 (3)	0.76507 (8)	0.0031 (2)	0.0191 (5)	
H10A	0.0374	0.7837	−0.0358	0.023*	
C11A	0.1047 (3)	0.71658 (8)	−0.0008 (2)	0.0221 (5)	
H11A	0.0167	0.7024	−0.0436	0.027*	
C12A	0.2235 (3)	0.68861 (8)	0.0597 (2)	0.0219 (5)	
H12A	0.2127	0.6557	0.0595	0.026*	
C13A	0.3581 (3)	0.70934 (8)	0.1204 (2)	0.0204 (5)	
H13A	0.4368	0.6904	0.1606	0.025*	
C14A	0.3744 (2)	0.75836 (8)	0.12070 (19)	0.0170 (4)	
S1B	0.70785 (7)	0.13290 (2)	0.26867 (5)	0.02190 (15)	
O1B	0.57264 (18)	0.46335 (6)	0.28156 (14)	0.0200 (3)	
N1B	0.6814 (2)	0.23011 (7)	0.26320 (16)	0.0162 (4)	
N2B	0.4813 (2)	0.19309 (7)	0.16560 (17)	0.0164 (4)	
N3B	0.4551 (2)	0.38927 (7)	0.17211 (16)	0.0169 (4)	
C1B	0.6236 (3)	0.18613 (8)	0.23241 (19)	0.0173 (4)	
C2B	0.5767 (2)	0.26526 (8)	0.21513 (18)	0.0146 (4)	
C3B	0.5805 (2)	0.31452 (8)	0.22219 (19)	0.0166 (4)	
H3BA	0.6648	0.3305	0.2649	0.020*	
C4B	0.4516 (2)	0.33902 (8)	0.16207 (19)	0.0158 (4)	
C5B	0.3242 (2)	0.31426 (8)	0.0986 (2)	0.0178 (5)	
H5BA	0.2401	0.3314	0.0592	0.021*	
C6B	0.3211 (2)	0.26541 (8)	0.09358 (19)	0.0180 (5)	
H6BA	0.2364	0.2492	0.0518	0.022*	
C7B	0.4495 (2)	0.24101 (8)	0.15326 (19)	0.0156 (4)	
C8B	0.3622 (3)	0.41641 (8)	0.10194 (19)	0.0178 (4)	
H8BA	0.2919	0.4027	0.0412	0.021*	
C9B	0.3649 (2)	0.46731 (8)	0.11564 (19)	0.0172 (4)	
C10B	0.2614 (3)	0.49637 (8)	0.0404 (2)	0.0213 (5)	
H10B	0.1878	0.4822	−0.0171	0.026*	
C11B	0.2659 (3)	0.54507 (9)	0.0494 (2)	0.0244 (5)	
H11B	0.1962	0.5637	−0.0013	0.029*	
C12B	0.3766 (3)	0.56628 (9)	0.1357 (2)	0.0238 (5)	
H12B	0.3814	0.5993	0.1413	0.029*	
C13B	0.4791 (3)	0.53897 (8)	0.2129 (2)	0.0204 (5)	
H13B	0.5519	0.5536	0.2702	0.024*	
C14B	0.4731 (2)	0.48965 (8)	0.20457 (19)	0.0162 (4)	
H1NA	0.475 (4)	1.0777 (10)	0.181 (3)	0.043 (9)*	
H2NA	0.145 (3)	1.0262 (9)	−0.038 (3)	0.033 (7)*	
H1NB	0.774 (3)	0.2355 (9)	0.302 (2)	0.026 (7)*	
H2NB	0.424 (3)	0.1704 (10)	0.139 (2)	0.031 (8)*	
H1OB	0.548 (4)	0.4299 (13)	0.259 (3)	0.077 (12)*	
H1OA	0.501 (4)	0.8131 (13)	0.168 (3)	0.075 (12)*	

### Atomic displacement parameters (Å^2^)

**Table:**

	*U*^11^	*U*^22^	*U*^33^	*U*^12^	*U*^13^	*U*^23^
S1A	0.0222 (3)	0.0178 (3)	0.0236 (3)	−0.0002 (2)	−0.0030 (2)	0.0006 (2)
O1A	0.0183 (8)	0.0214 (10)	0.0261 (8)	0.0010 (7)	−0.0065 (6)	−0.0010 (7)
N1A	0.0165 (8)	0.0158 (10)	0.0191 (9)	−0.0018 (7)	0.0002 (7)	−0.0006 (7)
N2A	0.0205 (9)	0.0166 (10)	0.0176 (9)	−0.0005 (8)	−0.0022 (7)	0.0003 (7)
N3A	0.0190 (9)	0.0185 (11)	0.0153 (8)	0.0003 (7)	0.0023 (7)	0.0005 (7)
C1A	0.0178 (10)	0.0195 (12)	0.0154 (9)	−0.0011 (9)	0.0035 (8)	−0.0015 (8)
C2A	0.0169 (10)	0.0176 (12)	0.0137 (9)	−0.0002 (8)	0.0025 (8)	0.0006 (8)
C3A	0.0169 (10)	0.0208 (13)	0.0173 (10)	−0.0014 (9)	−0.0007 (8)	−0.0001 (9)
C4A	0.0172 (10)	0.0169 (12)	0.0161 (10)	0.0002 (8)	0.0049 (8)	−0.0009 (8)
C5A	0.0158 (10)	0.0222 (13)	0.0185 (10)	0.0023 (9)	0.0003 (8)	0.0003 (9)
C6A	0.0161 (10)	0.0232 (13)	0.0177 (10)	−0.0006 (9)	−0.0001 (8)	−0.0007 (9)
C7A	0.0177 (10)	0.0162 (12)	0.0165 (9)	−0.0028 (8)	0.0037 (8)	−0.0007 (8)
C8A	0.0152 (9)	0.0225 (13)	0.0158 (10)	0.0029 (9)	0.0023 (8)	0.0012 (9)
C9A	0.0150 (10)	0.0178 (12)	0.0148 (9)	0.0013 (8)	0.0033 (8)	0.0011 (8)
C10A	0.0147 (9)	0.0202 (13)	0.0222 (11)	0.0007 (9)	0.0001 (8)	0.0008 (9)
C11A	0.0162 (10)	0.0242 (14)	0.0259 (11)	−0.0037 (9)	0.0006 (9)	−0.0013 (10)
C12A	0.0215 (11)	0.0163 (13)	0.0282 (12)	−0.0008 (9)	0.0042 (9)	0.0015 (9)
C13A	0.0182 (10)	0.0230 (13)	0.0200 (10)	0.0057 (9)	0.0002 (8)	0.0036 (9)
C14A	0.0157 (9)	0.0205 (12)	0.0149 (9)	−0.0010 (9)	0.0027 (8)	−0.0010 (8)
S1B	0.0239 (3)	0.0184 (3)	0.0230 (3)	0.0036 (2)	−0.0042 (2)	−0.0003 (2)
O1B	0.0208 (8)	0.0181 (9)	0.0208 (8)	−0.0001 (6)	−0.0044 (6)	0.0007 (6)
N1B	0.0153 (8)	0.0170 (10)	0.0162 (8)	0.0002 (7)	−0.0014 (7)	−0.0006 (7)
N2B	0.0165 (8)	0.0146 (10)	0.0180 (8)	−0.0011 (8)	0.0001 (7)	−0.0020 (7)
N3B	0.0171 (8)	0.0161 (10)	0.0176 (8)	0.0002 (7)	0.0028 (7)	−0.0003 (7)
C1B	0.0181 (10)	0.0197 (12)	0.0143 (9)	0.0007 (9)	0.0030 (8)	−0.0013 (8)
C2B	0.0135 (9)	0.0185 (12)	0.0118 (9)	0.0003 (8)	0.0019 (7)	−0.0009 (8)
C3B	0.0152 (9)	0.0198 (12)	0.0149 (9)	−0.0025 (8)	0.0010 (8)	−0.0026 (8)
C4B	0.0177 (10)	0.0150 (12)	0.0152 (9)	0.0005 (8)	0.0047 (8)	0.0001 (8)
C5B	0.0149 (9)	0.0189 (12)	0.0197 (10)	0.0011 (9)	0.0006 (8)	0.0016 (9)
C6B	0.0154 (10)	0.0202 (12)	0.0182 (10)	−0.0028 (8)	−0.0007 (8)	−0.0010 (9)
C7B	0.0162 (9)	0.0164 (12)	0.0142 (9)	−0.0007 (8)	0.0030 (8)	−0.0013 (8)
C8B	0.0200 (10)	0.0181 (12)	0.0154 (9)	−0.0016 (9)	0.0021 (8)	−0.0020 (8)
C9B	0.0187 (10)	0.0183 (12)	0.0148 (9)	−0.0006 (9)	0.0027 (8)	0.0000 (8)
C10B	0.0237 (11)	0.0217 (13)	0.0183 (10)	0.0011 (9)	−0.0016 (9)	0.0003 (9)
C11B	0.0299 (12)	0.0220 (14)	0.0213 (11)	0.0075 (10)	0.0003 (9)	0.0042 (9)
C12B	0.0313 (12)	0.0182 (13)	0.0221 (11)	0.0024 (10)	0.0053 (10)	0.0012 (9)
C13B	0.0219 (11)	0.0199 (13)	0.0194 (10)	−0.0016 (9)	0.0020 (9)	−0.0028 (9)
C14B	0.0164 (9)	0.0168 (12)	0.0156 (9)	0.0012 (8)	0.0044 (8)	0.0006 (8)

### Geometric parameters (Å, °)

**Table:**

S1A—C1A	1.684 (2)	S1B—C1B	1.688 (2)
O1A—C14A	1.360 (2)	O1B—C14B	1.351 (2)
O1A—H1OA	0.99 (4)	O1B—H1OB	0.99 (4)
N1A—C1A	1.359 (3)	N1B—C1B	1.359 (3)
N1A—C7A	1.391 (3)	N1B—C2B	1.395 (3)
N1A—H1NA	0.86 (3)	N1B—H1NB	0.87 (2)
N2A—C1A	1.353 (3)	N2B—C1B	1.360 (3)
N2A—C2A	1.388 (3)	N2B—C7B	1.377 (3)
N2A—H2NA	0.86 (3)	N2B—H2NB	0.84 (3)
N3A—C8A	1.291 (3)	N3B—C8B	1.293 (3)
N3A—C4A	1.420 (3)	N3B—C4B	1.416 (3)
C2A—C3A	1.374 (3)	C2B—C3B	1.387 (3)
C2A—C7A	1.405 (3)	C2B—C7B	1.392 (3)
C3A—C4A	1.402 (3)	C3B—C4B	1.398 (3)
C3A—H3AA	0.9300	C3B—H3BA	0.9300
C4A—C5A	1.408 (3)	C4B—C5B	1.408 (3)
C5A—C6A	1.387 (3)	C5B—C6B	1.374 (3)
C5A—H5AA	0.9300	C5B—H5BA	0.9300
C6A—C7A	1.381 (3)	C6B—C7B	1.392 (3)
C6A—H6AA	0.9300	C6B—H6BA	0.9300
C8A—C9A	1.447 (3)	C8B—C9B	1.438 (3)
C8A—H8AA	0.9300	C8B—H8BA	0.9300
C9A—C10A	1.405 (3)	C9B—C10B	1.404 (3)
C9A—C14A	1.409 (3)	C9B—C14B	1.415 (3)
C10A—C11A	1.368 (3)	C10B—C11B	1.372 (3)
C10A—H10A	0.9300	C10B—H10B	0.9300
C11A—C12A	1.393 (3)	C11B—C12B	1.396 (3)
C11A—H11A	0.9300	C11B—H11B	0.9300
C12A—C13A	1.388 (3)	C12B—C13B	1.383 (3)
C12A—H12A	0.9300	C12B—H12B	0.9300
C13A—C14A	1.384 (3)	C13B—C14B	1.390 (3)
C13A—H13A	0.9300	C13B—H13B	0.9300
			
C14A—O1A—H1OA	108 (2)	C14B—O1B—H1OB	105 (2)
C1A—N1A—C7A	110.31 (18)	C1B—N1B—C2B	110.49 (17)
C1A—N1A—H1NA	125 (2)	C1B—N1B—H1NB	124.5 (16)
C7A—N1A—H1NA	125 (2)	C2B—N1B—H1NB	124.8 (16)
C1A—N2A—C2A	110.80 (18)	C1B—N2B—C7B	110.32 (18)
C1A—N2A—H2NA	122.8 (18)	C1B—N2B—H2NB	122.1 (19)
C2A—N2A—H2NA	125.0 (18)	C7B—N2B—H2NB	127.5 (19)
C8A—N3A—C4A	121.14 (18)	C8B—N3B—C4B	122.41 (18)
N2A—C1A—N1A	106.66 (19)	N1B—C1B—N2B	106.32 (18)
N2A—C1A—S1A	125.40 (16)	N1B—C1B—S1B	127.87 (16)
N1A—C1A—S1A	127.91 (17)	N2B—C1B—S1B	125.81 (17)
C3A—C2A—N2A	131.86 (19)	C3B—C2B—C7B	121.93 (19)
C3A—C2A—C7A	122.13 (19)	C3B—C2B—N1B	132.46 (18)
N2A—C2A—C7A	106.01 (18)	C7B—C2B—N1B	105.60 (19)
C2A—C3A—C4A	117.38 (19)	C2B—C3B—C4B	116.88 (18)
C2A—C3A—H3AA	121.3	C2B—C3B—H3BA	121.6
C4A—C3A—H3AA	121.3	C4B—C3B—H3BA	121.6
C3A—C4A—C5A	120.2 (2)	C3B—C4B—C5B	120.9 (2)
C3A—C4A—N3A	124.00 (19)	C3B—C4B—N3B	116.39 (18)
C5A—C4A—N3A	115.81 (18)	C5B—C4B—N3B	122.72 (18)
C6A—C5A—C4A	122.0 (2)	C6B—C5B—C4B	121.63 (19)
C6A—C5A—H5AA	119.0	C6B—C5B—H5BA	119.2
C4A—C5A—H5AA	119.0	C4B—C5B—H5BA	119.2
C7A—C6A—C5A	117.28 (19)	C5B—C6B—C7B	117.51 (19)
C7A—C6A—H6AA	121.4	C5B—C6B—H6BA	121.2
C5A—C6A—H6AA	121.4	C7B—C6B—H6BA	121.2
C6A—C7A—N1A	132.74 (19)	N2B—C7B—C6B	131.55 (19)
C6A—C7A—C2A	121.0 (2)	N2B—C7B—C2B	107.26 (18)
N1A—C7A—C2A	106.21 (18)	C6B—C7B—C2B	121.2 (2)
N3A—C8A—C9A	121.52 (19)	N3B—C8B—C9B	121.54 (19)
N3A—C8A—H8AA	119.2	N3B—C8B—H8BA	119.2
C9A—C8A—H8AA	119.2	C9B—C8B—H8BA	119.2
C10A—C9A—C14A	118.4 (2)	C10B—C9B—C14B	118.0 (2)
C10A—C9A—C8A	119.47 (19)	C10B—C9B—C8B	121.02 (19)
C14A—C9A—C8A	122.10 (19)	C14B—C9B—C8B	120.99 (19)
C11A—C10A—C9A	121.2 (2)	C11B—C10B—C9B	121.8 (2)
C11A—C10A—H10A	119.4	C11B—C10B—H10B	119.1
C9A—C10A—H10A	119.4	C9B—C10B—H10B	119.1
C10A—C11A—C12A	119.6 (2)	C10B—C11B—C12B	119.0 (2)
C10A—C11A—H11A	120.2	C10B—C11B—H11B	120.5
C12A—C11A—H11A	120.2	C12B—C11B—H11B	120.5
C13A—C12A—C11A	120.7 (2)	C13B—C12B—C11B	121.0 (2)
C13A—C12A—H12A	119.6	C13B—C12B—H12B	119.5
C11A—C12A—H12A	119.6	C11B—C12B—H12B	119.5
C14A—C13A—C12A	119.7 (2)	C12B—C13B—C14B	119.8 (2)
C14A—C13A—H13A	120.2	C12B—C13B—H13B	120.1
C12A—C13A—H13A	120.2	C14B—C13B—H13B	120.1
O1A—C14A—C13A	118.99 (19)	O1B—C14B—C13B	119.24 (19)
O1A—C14A—C9A	120.7 (2)	O1B—C14B—C9B	120.5 (2)
C13A—C14A—C9A	120.29 (19)	C13B—C14B—C9B	120.29 (19)

### Hydrogen-bond geometry (Å, °)

**Table:**

*D*—H···*A*	*D*—H	H···*A*	*D*···*A*	*D*—H···*A*
N1A—H1NA···S1B^i^	0.86 (3)	2.61 (3)	3.4714 (19)	173 (3)
N2A—H2NA···O1B^ii^	0.86 (3)	1.99 (3)	2.781 (2)	153 (3)
N1B—H1NB···O1A^iii^	0.87 (2)	2.16 (2)	2.936 (2)	149 (2)
N2B—H2NB···S1A^iv^	0.84 (3)	2.45 (3)	3.2547 (19)	163 (2)
O1B—H1OB···N3B	0.99 (4)	1.64 (3)	2.552 (2)	152 (3)
O1A—H1OA···N3A	0.99 (4)	1.72 (4)	2.600 (3)	147 (3)

Symmetry codes: (i) *x*, *y*+1, *z*; (ii) *x*−1/2, −*y*+3/2, *z*−1/2; (iii) −*x*+3/2, *y*−1/2, −*z*+1/2; (iv) *x*, *y*−1, *z*.
